# Appropriate Body Mass Index and Waist Circumference Cutoffs for Categorization of Overweight and Central Adiposity among Uighur Adults in Xinjiang

**DOI:** 10.1371/journal.pone.0080185

**Published:** 2013-11-07

**Authors:** Shuo Pan, Zi-Xiang Yu, Yi-Tong Ma, Fen Liu, Yi-Ning Yang, Xiang Ma, Zhen-Yan Fu, Xiao-Mei Li, Xiang Xie, You Chen, Bangdang Chen, Chun-Hui He

**Affiliations:** 1 Department of Cardiology, First Affiliated Hospital of Xinjiang Medical University, Urumqi, People’s Republic of China; 2 Xinjiang Key Laboratory of Cardiovascular Disease Research, Urumqi, People’s Republic of China; University of Louisville, United States of America

## Abstract

**Objective:**

The current overweight and central adiposity guidelines based on Western populations were not consistent with many studied based on the Asian populations. Uighur people live in Xinjiang Uighur Autonomous Region which is located in the center of Asia. Their overweight and central cutoffs were largely unknown. We aimed to identify cutoffs for body mass index (BMI; in kg/m^2^) and waist circumference (WC; in cm) for categorization of overweight and central adiposity among Uighur adults in Xinjiang.

**Methods:**

4767 Uighur participants were selected from the Cardiovascular Risk Survey (CRS) which was carried out from October 2007 to March 2010. The age of the participants were from 35 to 101 years old with the mean age of 50.09 years. Anthropometric data, blood pressure, serum concentration of serum total cholesterol, triglyceride, low density lipoprotein (LDL), high density lipoprotein (HDL) and fasting glucose were documented. The prevalence, sensitivity, specificity and distance on the receiver operating characteristic (ROC) curve of each BMI and waist circumference values were calculated.

**Results:**

The prevalence of hypertension, hypercholesterolemia and hypertriglyceridemia were higher with higher BMI for both men and women. The prevalence of hypertension and hypercholesterolemia were higher with higher waist circumference for both men and women. In women, the prevalence of hypertriglyceridemia was noticed to increase as the waist circumference increased. The shortest distance in the receiver operating characteristic curves for hypertension, dyslipidemia, diabetes, or ≥ 2 of these risk factors suggested a BMI cutoff of 26 and a waist circumference cutoff of 90 cm for both men and women.

**Conclusions:**

Higher cutoffs for BMI and waist circumference are needed in the identification of Uighur patients at high risk of cardiovascular disease.

## Introduction

Obesity has been increasing in epidemic proportions in both adults and children [1,2]. The prevalence among adults had increased by nearly 50% from the 1980s to 1990s [3]. Now, nearly 70% of adults are classified as overweight or obese compared with 25% 40 years ago [4,5]. Additionally, it was found that the proportion of the population the morbid obesity had increased by a greater extent than overweight and mild obesity [5]. A recent study of nearly 360,000 participants from 9 European countries showed that both general obesity and abdominal adiposity were associated with risk of death and it supported the importance of WC and BMI for assessing mortality risks [6]. Obesity has also been shown to be associated with numerous cardiovascular disease risk factors, such as hypertension, dyslipidemia, type 2 diabetes and insulin resistance [7,8].

In 1998, WHO announced that the definition of overweight and central adiposity as body mass index (BMI, in kg/m^2^) ≥ 25 and waist circumference (WC) ≥ 94 cm (Asian ≥ 90 cm) for men and ≥ 80cm for women [9]. Some literatures indicated that the cutoffs for both men and women could be lower among Asian populations [10,11]. Several epidemiologic studies in Asian populations have shown that Asians have higher amounts of body fat at lower BMIs and WCs than the Western populations [12-14], it may lead to the greater prevalence of cardiovascular disease risk factors at lower BMIs and WCs in Asian populations than in Western populations [15–18]. 

The overweight in Asia-Pacific region was defined as a BMI ≥ 23 and the WC cutoffs were 90 cm for men and 80 cm for women [19]. The study based on Chinese population suggested that a BMI cutoff of 24 and a WC cutoff of 80 cm for both men and women [20]. The study based on Korean adults showed that the appropriate WC cutoff point for central obesity in Koreans was determined to be 90 cm for men and 85 cm for women [21]. A recent Thailand study showed that the appropriate cutoff point of BMI was 23 kg/m^2^ in men and 24 kg/m^2^ in women. The cutoffs of WC were 80 cm and 78 cm in men and women, respectively [22].

 The data above suggested even in Asian population, the BMI and the WC cutoffs may be different. It was important to apply the ethnically appropriate cutoff values of BMI and WC for assessing overweight and central obesity. In Xinjiang Uighur Autonomous Region northeast of China, the dominant ethnic was Uighur population. In this study, we investigated the BMI and the WC cutoffs in Uighur population. 

## Methods

### Ethics Statement

This study was approved by the Ethics Committee of the First Affiliated Hospital of Xinjiang Medical University and was conducted according to the standards of the Declaration of Helsinki. Written, informed consent was obtained from the participants.

### Sample design

All the participants were selected from the Cardiovascular Risk Survey (CRS) study, the detailed description of the study population and the methods were described previously [23,24]. Briefly, the CRS study used a 4-stage stratified sampling method to select a representative sample of the general population in Xinjiang, northwest of China. The research sites included Urumqi City, Kelamayi City, Fukang City, Turpan Prefecture, Hetian Prefecture, Yili Prefecture. The time period was from October 2007 to March 2010. The selections made from sampling units were based on geographic area, sex, and age groups using household registries. The 4-stage stratified sampling method was as follows: Stage one, according to population census data of Xinjiang in 2000, the area mentioned above were selected based on population, ethnicity, geography, economic and cultural development level respectively. Stage two, according to the ethnic aggregation status, one district or county was randomly selected from the Uighur population dominated area. Stage three, one community or town (village) was randomly selected from each district or county. Stage four, sujects aged above 35 years were randomly selected from each community or town (village) as research subjects. The staff conducted surveys in households and administered questionnaires. The questionnaires included the demographic, socioeconomic, dietary, and medical history of each participant. In total, the CRS included 14 618 participants (5757 Hans, 4767 Uighurs, and 4094 Kazakhs). 

4687 Uighur participants with complete data were enrolled in the present study. 1983 participants were male and 2704 participants were female. The age of the participants were from 35 to 101 years old with the mean ± SD age of 50.09 ± 13.01 (men, 52.62 ± 13.58; women, 49.28 ± 12.38).

### Anthropometric measurements

Data collection involved one visit in the participants’ residential areas. During the examinations, a standard questionnaire assessing demographic information and medical history was collected by trained research staff. Body weight was measured while the subjects were without clothes or shoes by using a double balance placed on a firm surface. Height was measured by using a Frankfort place positioned at a 90° angle against a wall-mounted metal tape. The WC measurements were taken at the end of normal expiration and to the nearest 0.1 cm, measuring from the narrowest point between the lower borders of the rib cage and the iliac crest. BMI was calculated as weight in kg divided by height in m^2^.

### Laboratory methods

Blood samples were obtained from an antecubital vein into vacutainer tubes containing EDTA in the morning after an overnight fasting period. All the collected samples were transported on dry ice at prearranged intervals to Xinjiang coronary artery disease VIP laboratory. The serum concentration of serum total cholesterol, triglyceride, low density lipoprotein (LDL), high density lipoprotein (HDL) and fasting glucose were measured by the Clinical Laboratory Department of the First Affiliated Hospital of Xinjiang Medical University with the biochemical analyzer (Dimension AR/AVL Clinical Chemistry System, Newark, NJ, USA) [25,26].

### Blood pressure measurement

A mercury sphygmomanometer was used to measure the blood pressure in the sitting position after a 10 min rest period. During the 30 min preceding the measurement, the subjects were required to refrain from smoking or consuming caffeine. The appearance of the first sound was used to define systolic blood pressure, and the disappearance of sound was used to define diastolic blood pressure [27]. Two readings each of systolic and diastolic blood pressures were recorded, and the average of each measurement was used for data analysis. If the first two measurements differed by more than 5 mmHg, additional readings were taken.

### Definition of risk factors

Hypertension was defined as self-reported use of antihypertensive medication within the past 2 week or an average systolic blood pressure ≥ 140 mm Hg, an average diastolic blood pressure ≥ 90 mm Hg, or both.

Diabetes was defined as fasting plasma glucose ≥ 7.0mmol/L, use of insulin or oral hypoglycemic agents, or a self-reported history of diabetes.

Total cholesterol concentration > 6.22 mmol/L (240 mg/dl) was defined as Hypercholesterolemia, triglyceride concentration > 2.26 mmol/L (200 mg/dl) was defined as hypertriglyceridemia. LDL cholesterol concentration > 4.14 mmol/L (160 mg/dl) was defined as high LDL cholesterol. HDL cholesterol concentration < 1.04 mmol/L (40 mg/dl) was defined as low HDL cholesterol [28]. Dislipidemia was defined as anyone of the four lipids abnormalities above or self-reported use of antihyperlipidemic medication. 

Definition of overweight and obesity in adults using BMI were as follows: 25-29.9—overweight, over 30—obesity [9].

Definition of central adiposity in adults using WC was as follows: Men: 94 cm (Asian men 90 cm) or more; Women: 80 cm or more [9,29].

### Statistical analysis

The statistical analysis was conducted using SPSS version 16.0 for Windows (SPSS Inc., Chicago, IL, USA). Continuous variables were expressed as gender specific means and standard deviations, and discrete variables were expressed as gender-specific proportions. Age-standardization was performed by the direct method by using the 2000 Xinjiang Uighur population as the standard population. The sensitivity and specificity of each BMI and waist circumference level for the detection of hypertension, dyslipidemia, diabetes, and 2 or more of these risk factors were calculated by creating dichotomous variables for each BMI and waist circumference value. Additionally, the distance on the receiver operating characteristic (ROC) curve of each BMI and waist circumference value was calculated as the square root of [(1 - sensitivity)^2^ + (1 - specificity)^2^]. The BMI or waist circumference value with the shortest distance on the ROC curve was considered in the determination of appropriate cutoffs. 

## Results

### Body mass index

For both men and women, the systolic blood pressure, diastolic blood pressure increased with the increase of BMI. We also noticed the triglycerides increased with the increase of BMI in men. We did not notice the trend in other values in both men and women (Table **1**).

**Table 1:** Age-standardized cardiovascular disease risk factors in the Uighur population by BMI category.

	BMI<21	21≤BMI<23	23≤BMI<25	25≤BMI<27	27≤BMI<29	BMI≥29	*P* value
**Men**							
Population distribution (%)	262 (13.2%)	321 (16.2%)	377 (19.0%)	339 (17.1%)	312 (15.7%)	372 (18.85%)	
Systolic blood pressure (mmHg)	121.62±16.62	128.08±19.42	130.02±17.86	131.96±19.52	136.17±20.21	138.44±19.73	<0.001
Diastolic blood pressure (mmHg)	73.58±12.34	77.45±13.35	78.88±13.19	81.90±15.07	84.32±14.43	85.17±14.95	<0.001
Total cholesterol (mmol/L)	3.86±0.90	3.98±0.90	4.28±1.06	4.41±1.07	4.56±1.35	4.55±1.01	<0.001
HDL cholesterol (mmol/L)	1.29±0.40	1.30±0.46	1.27±0.47	1.24±0.48	1.21±0.40	1.26±0.64	0.170
LDL cholesterol (mmol/L)	2.91±0.90	2.95±0.91	2.82±0.87	2.93±0.98	2.83±0.95	2.80±0.86	0.156
Triglycerides (mmol/L)	1.06±0.56	1.21±0.70	1.51±0.91	1.80±1.40	1.99±1.54	2.27±1.74	<0.001
Fasting glucose (mmol/L)	4.56±0.95	4.84±1.87	4.86±1.54	5.12±2.24	4.94±1.76	5.25±2.17	<0.001
**Women**							
Population distribution (%)	357 (13.2%)	368 (13.6%)	446 (16.5%)	449 (16.6%)	403 (14.9%)	681 (25.2%)	
Systolic blood pressure (mmHg)	122.69±19.98	123.73±18.83	127.54±20.40	131.36±22.15	135.87±22.03	140.01±22.72	<0.001
Diastolic blood pressure (mmHg)	73.72±13.45	75.47±12.99	78.63±15.11	80.27±15.18	82.30±15.03	84.04±15.28	<0.001
Total cholesterol (mmol/L)	4.00±0.95	4.29±1.23	4.25±1.04	4.46±1.07	4.58±1.14	4.64±1.26	<0.001
HDL cholesterol (mmol/L)	1.26±0.38	1.32±0.42	1.22±0.42	1.24±0.52	1.25±0.44	1.26±0.47	0.084
LDL cholesterol (mmol/L)	2.90±0.88	2.93±0.88	2.83±0.94	2.82±0.94	2.85±0.93	2.85±0.96	0.529
Triglycerides (mmol/L)	1.18±0.80	1.25±0.80	1.49±1.02	1.71±1.06	1.92±1.50	1.90±1.25	<0.001
Fasting glucose (mmol/L)	4.73±1.34	4.71±1.33	4.89±1.54	4.98±1.63	5.09±1.68	5.09±1.53	<0.001

Continuous variables are expressed as mean ± s.d. Categorical variables are expressed as percentages.

Likewise, when continuous risk factors were categorized, the prevalence of hypertension and hypercholesterolemia were higher with higher BMIs for both men and women. In women, the prevalence of hypertriglyceridemia appeared to be higher with higher BMIs. The prevalence of diabetes, high LDL cholesterol and low HDL cholesterol did not show the apparent trend (Table **2**).

**Table 2:** Age-standardized prevalence of risk factors in the Uighur population by BMI category.

	BMI<21	21≤BMI<23	23≤BMI<25	25≤BMI<27	27≤BMI<29	BMI≥29	*P* value
**Men**							
Hypertension	16.5%	27.4%	29.4%	32.7%	37.1%	44.4%	<0.001
Diabetes	2.8%	5.8%	4.5%	8.2%	5.0%	10.8%	<0.001
Hypercholesterolemia	10.3%	14.1%	29.7%	37.9%	43.5%	53.5%	<0.001
High LDL cholesterol	40.1%	42.4%	35.8%	42.6%	33.0%	33.2%	0.021
Low HDL cholesterol	27.8%	29.9%	30.6%	35.0%	34.0%	34.9%	0.283
Hypertriglyceridemia	5.9%	8.1%	17.4%	18.7%	23.7%	20.7%	<0.001
**Women**							
Hypertension	19.7%	22.4%	27.8%	33.6%	38.2%	47.5%	<0.001
Diabetes	4.0%	2.5%	4.4%	6.8%	6.1%	8.2%	0.002
Hypercholesterolemia	15.6%	17.5%	25.7%	39.0%	43.7%	45.2%	<0.001
High LDL cholesterol	41.9%	34.6%	37.0%	35.0%	35.4%	35.1%	0.304
Low HDL cholesterol	29.2%	28.2%	36.0%	35.5%	32.7%	31.6%	0.101
Hypertriglyceridemia	8.4%	14.2%	15.3%	22.8%	24.2%	25.9%	<0.001

The population percentile of each BMI level and the sensitivity, specificity, and distance on the ROC curve for the detection of hypertension, dyslipidemia, diabetes, and ≥ 2 of these risk factors were presented in Table **3** for men and women separately. In both men and women, the specificity, sensitivity, and distances on the ROC curves were similar for all 3 cardiovascular disease risk factors. Except that the cutoff for dislipidemia in men was 25, the shortest distance on the ROC curve of hypertension and diabetes in both men and women and the shortest distance on the ROC curve of dyslipidemia in women were the same, a BMI of 26 appeared to be the optimal BMI cutoffs in both men and women. The shortest distance on the ROC curve of ≥ 2 risk factors was also at a BMI of 26 for men and women.

**Table 3:** Sensitivity (Sens), specificity (Spec), and distance in the receiver operating characteristic (ROC) curve for BMI cutoffs.

		Hypertension			Dislipidemia			Diabetes			≥2 risk factoes		
BMI cutoffs (kg/m^2^)	Percentile	Sens	Spec	Distance in ROC curve	Sens	Spec	Distance in ROC curve	Sens	Spec	Distance in ROC curve	Sens	Spec	Distance in ROC curve
**Men**		%			%			%			%		
22	20.4%	87.0	25.0	0.76	91.8	29.4	0.71	86.0	21.7	0.80	94.0	24.4	0.76
23	29.4%	78.3	34.3	0.69	86.2	40.5	0.61	78.1	30.7	0.73	88.5	34.1	0.67
24	38.7%	70.1	42.9	0.65	78.3	49.9	0.55	71.5	39.7	0.67	81.3	43.2	0.60
25	48.6%	61.7	53.1	0.61	69.0	59.2	**0.51**	63.7	49.1	0.63	73.0	52.8	0.54
26	57.6%	53.6	62.6	**0.60**	59.3	67.9	0.52	54.2	58.2	**0.62**	64.4	62.0	**0.52**
27	65.5%	44.5	71.2	0.63	48.9	75.9	0.56	45.1	67.1	0.64	54.8	70.8	0.54
28	74.7%	35.0	81.1	0.68	38.3	83.0	0.64	37.2	75.5	0.67	45.0	78.9	0.59
29	81.2%	26.4	84.7	0.75	29.1	87.9	0.72	31.4	82.2	0.71	36.2	85.0	0.66
30	85.8%	18.8	88.8	0.82	21.8	91.6	0.79	24.8	87.2	0.76	27.8	89.3	0.73
**Women**													
22	19.0%	88.9	23.0	0.78	90.4	25.8	0.75	89.4	19.7	0.81	93.9	22.5	0.78
23	26.8%	83.4	31.6	0.70	85.1	34.8	0.69	85.4	27.4	0.74	89.7	30.9	0.70
24	34.3%	76.6	41.1	0.63	78.4	44.4	0.60	78.8	35.9	0.68	83.4	39.8	0.62
25	43.3%	69.5	50.1	0.58	71.0	53.5	0.55	71.2	44.3	0.63	77.7	48.8	0.56
26	51.7%	61.4	58.3	**0.57**	62.2	61.2	**0.54**	61.3	52.3	**0.61**	70.4	57.3	**0.52**
27	59.9%	52.7	66.3	0.58	52.5	68.2	0.54	52.7	60.4	0.62	61.1	65.1	0.52
28	68.4%	43.7	73.9	0.62	42.3	74.8	0.63	44.9	68.6	0.63	51.1	72.6	0.56
29	78.4%	35.4	80.0	0.68	33.2	80.3	0.70	35.5	75.4	0.69	41.5	79.0	0.62
30	81.3%	28.4	85.6	0.73	25.7	85.3	0.76	26.5	81.2	0.76	33.2	84.3	0.69

### Waist circumference

For men, the systolic blood pressure, diastolic blood pressure and triglycerides increased with the increase of waist circumference. We did not notice the trend in total cholesterol, LDL-cholesterol, HDL-cholesterol and fasting glucose values. For women, the systolic blood pressure, diastolic blood pressure, total cholesterol and triglycerides increased with the increase of waist circumference, the LDL-cholesterol, HDL-cholesterol and fasting glucose did not show the trend (Table **4**).

**Table 4:** Age-standardized mean cardiovascular disease risk factors in the Uighur population by waist circumference category.

	Waist circumference (cm)						
	<75	75-79.9	80-84.9	85-89.9	90-94.9	≥95	*P* value
**Men**							
Population distribution (%)	275 (13.9%)	181 (9.1%)	272 (13.7%)	295 (14.9%)	315 (15.9%)	645 (32.5%)	
Systolic blood pressure (mmHg)	122.38±15.89	124.66±16.43	128.51±18.88	130.48±17.83	133.49±20.96	137.98±20.10	<0.001
Diastolic blood pressure (mmHg)	73.72±10.92	76.06±12.92	77.50±13.88	80.24±13.26	82.01±15.39	85.29±14.82	<0.001
Total cholesterol (mmol/L)	3.95±1.02	3.89±0.84	4.15±0.92	4.25±1.12	4.53±1.18	4.52±1.11	<0.001
HDL cholesterol (mmol/L)	1.29±0.43	1.28±0.46	1.25±0.42	1.30±0.43	1.26±0.45	1.23±0.59	0.386
LDL cholesterol (mmol/L)	2.92±0.86	2.82±0.96	2.93±0.95	2.87±0.89	2.91±0.90	2.82±0.92	0.399
Triglycerides (mmol/L)	1.10±0.63	1.20±0.79	1.37±0.83	1.51±1.09	1.86±1.39	2.15±1.64	<0.001
Fasting glucose (mmol/L)	4.70±1.82	4.62±1.10	4.61±0.97	4.87±1.47	5.08±2.12	5.25±2.22	<0.001
**Women**							
Population distribution (%)	402 (14.9%)	331 (12.2%)	358 (13.2%)	416 (15.4%)	382 (14.1%)	815 (30.1%)	
Systolic blood pressure (mmHg)	121.00±20.07	123.16±18.66	125.92±19.97	130.24±19.97	136.22±21.38	140.56±22.83	<0.001
Diastolic blood pressure (mmHg)	72.97±13.58	75.79±13.68	76.65±13.27	79.88±14.54	83.01±15.10	84.39±15.45	<0.001
Total cholesterol (mmol/L)	4.02±1.06	4.25±1.11	4.32±1.11	4.41±1.14	4.55±1.35	4.63±1.08	<0.001
HDL cholesterol (mmol/L)	1.30±0.41	1.25±0.42	1.25±0.48	1.27±0.45	1.26±0.50	1.24±0.44	0.383
LDL cholesterol (mmol/L)	2.89±0.87	2.82±0.90	2.83±0.86	2.86±0.94	2.93±0.96	2.84±0.97	0.546
Triglycerides (mmol/L)	1.13±0.79	1.31±0.93	1.41±0.81	1.59±0.99	1.80±1.36	2.01±1.34	<0.001
Fasting glucose (mmol/L)	4.64±1.24	4.74±1.39	4.90±1.42	4.79±1.16	4.97±1.50	5.24±1.86	<0.001

Continuous variables are expressed as mean ± s.d. Categorical variables are expressed as percentages.

When continuous risk factors were categorized, the prevalence of hypertension and hypercholesterolemia were higher with higher waist circumference for both men and women. In women, the hypertriglyceridemia was noticed to increase as the waist circumference increased (Table **5**).

**Table 5:** Age-standardized prevalence of risk factors in the Chinese population by waist circumference category.

	Waist circumference (cm)						
	<75	75-79.9	80-84.9	85-89.9	90-94.9	≥95	*P* value
**Men**							
Hypertension	15.5%	20.6%	28.9%	30.9%	35.0%	42.5%	<0.001
Diabetes	4.2%	2.8%	3.1%	6.3%	6.0%	9.9%	<0.001
Hypercholesterolemia	11.4%	14.8%	21.7%	25.9%	38.7%	51.4%	<0.001
High LDL cholesterol	39.3%	39.5%	40.6%	39.1%	38.8%	34.0%	0.362
Low HDL cholesterol	29.2%	30.5%	33.8%	27.9%	30.6%	36.0%	0.137
Hypertriglyceridemia	10.2%	6.8%	12.8%	16.0%	23.2%	19.4%	<0.001
**Women**							
Hypertension	16.8%	21.5%	25.7%	30.8%	39.5%	48.4%	<0.001
Diabetes	2.8%	3.1%	4.6%	3.7%	5.4%	9.8%	<0.001
Hypercholesterolemia	13.9%	17.9%	25.6%	32.5%	38.1%	49.7%	<0.001
High LDL cholesterol	36.4%	37.4%	37.2%	35.7%	36.1%	35.8%	0.994
Low HDL cholesterol	30.5%	32.1%	34.0%	31.1%	35.9%	31.7%	0.613
Hypertriglyceridemia	8.8%	14.2%	18.1%	18.6%	23.2%	26.1%	<0.001

The population percentile of each waist circumference level and the sensitivity, specificity, and distance on the ROC curve for the detection of hypertension, dyslipidemia, diabetes, and ≥ 2 of these risk factors are presented in Table **6** for men and women separately. In both men and women, the specificity, sensitivity, and distances on the ROC curves were similar for all 3 cardiovascular disease risk factors. The shortest distance on the ROC curve of hypertension, dyslipidemia and diabetes were the same, a waist circumference of 90 appeared to be the optimal waist circumference cutoffs in both men and women. The shortest distance on the ROC curve of ≥ 2 risk factors was also at a waist circumference of 90 for men and women.

**Table 6:** Sensitivity (Sens), specificity (Spec), and distance in the receiver operating characteristic (ROC) curve for waist circumference cutoffs.

		Hypertension			Dislipidemia			Diabetes			≥2 risk factoes		
WC cutoffs (cm)	Percentile	Sens	Spec	Distance in ROC curve	Sens	Spec	Distance in ROC curve	Sens	Spec	Distance in ROC curve	Sens	Spec	Distance in ROC curve
**Men**		%			%			%			%		
65	5.3%	98.7	2.2	0.98	99.2	2.7	0.97	98.8	2.1	0.98	99.0	2.2	0.98
70	13.8%	97.2	6.7	0.93	97.5	7.8	0.92	97.5	6.0	0.94	98.2	6.5	0.94
75	22.9%	92.9	17.7	0.83	93.3	19.5	0.81	90.1	14.9	0.86	95.0	16.5	0.84
80	36.6%	86.5	29.2	0.72	88.1	32.3	0.69	85.6	25.0	0.76	91.9	27.8	0.73
85	51.5%	73.6	43.4	0.62	78.3	48.1	0.56	78.1	39.0	0.65	83.5	42.6	0.60
90	66.4%	59.2	58.8	**0.58**	65.1	64.6	**0.50**	63.3	54.3	**0.51**	70.9	58.3	**0.51**
95	82.3%	41.3	73.7	0.64	45.4	78.2	0.59	49.2	70.3	0.59	50.4	73.3	0.56
100	91.6%	23.4	85.3	0.78	25.7	88.2	0.75	31.0	83.8	0.71	30.4	85.6	0.71
**Women**													
65	7.2%	99.0	3.7	0.96	98.8	3.9	0.96	96.4	2.7	0.97	99.5	3.4	0.97
70	14.9%	97.2	9.8	0.90	96.9	10.5	0.90	94.7	7.6	0.93	98.4	9.0	0.91
75	27.1%	93.1	19.3	0.81	93.4	21.3	0.79	92.7	15.7	0.85	96.7	18.1	0.82
80	40.3%	84.5	34.2	0.68	84.9	37.0	0.65	85.1	28.8	0.73	90.3	32.7	0.68
85	55.7%	74.1	49.3	0.57	73.0	51.7	0.55	74.5	42.5	0.63	82.0	47.6	0.55
90	69.9%	59.1	65.1	**0.54**	57.1	66.4	**0.54**	64.9	58.1	**0.55**	67.9	63.1	**0.49**
95	82.8%	43.0	77.9	0.61	41.1	78.8	0.63	50.7	71.9	0.57	52.1	76.6	0.53
100	91.2%	26.7	89.1	0.74	23.9	88.9	0.77	31.8	84.6	0.70	32.4	87.9	0.69

## Discussion

The total Uighur population was 8.4 million in 2000, 99.4% of the total population of Uighurs live in Xinjiang Uighur Autonomous Region which is located in the center of Asia. According to the WHO cutoffs for the designation of overweight and central adiposity [7], the present study showed that 51.4% of the male Uighur participants had a BMI ≥ 25 and 56.7 % of the female Uighur participant had a BMI ≥ 25. 33.6 % of the male Uighur participant had the WC > 90 cm and 59.7 % of the female Uighur participant had the WC > 80 cm. One study including a total of 15 838 Chinese individuals showed that the only 26.6% of male participants had BMI ≥ 25 and 31.8% of female participants had BMI ≥ 25. Similarly, 14.4% of male participants had the WC > 90 cm and 35.6% of female participants had the WC > 80 cm. The prevalence of overweight had reached 64.5% in the U.S. population [5], the prevalence of overweight and central adiposity in Uighur population was closer with that in the western population. Besides of the ethnic difference, the main reason may be the diet difference between the Chinese Han population and Uighur population, the Uighur population consumed more pasta, meat and milk products than the Han population did. Therefore, the prevalence of overweight and central adiposity in Uighur population was higher than that in the Chinese Han population.

Additionally, this study showed an increasing trend in prevalence of hypertension, hypercholesterolemia and hypertriglyceridemia with higher BMIs and WCs among Uighur adults. Based on the sensitivity, specificity, and ROC calculations, this present study suggested a BMI of 26 and a WC of 90 cm for both men and women as more appropriate cutoffs for the designation of overweight and central adiposity in the Uighur population.

According to the WHO definition of obesity, overweight and central adiposity using BMI and WC cutoff values in adults [9,29], our results showed slightly higher BMI cutoffs than WHO cutoffs for the definition of overweight. The WC cut-off values for Uighur men were consistent with the WHO definition. However, we found the WC cut-off values for Uighur woman were much higher than WHO definition.

Since the joint WHO/IASO/IOTF report, several studies have examined appropriate cutoffs for overweight in Asian populations. The cutoffs which was proposed to define overweight and central adiposity were all lower than those which WHO recommended, they were also lower than those recommended by the joint WHO/ IASO/IOTF committee for central adiposity (90 cm for men and 80 cm for women) (19). Most studies suggested a BMI cutoff of 22–24 for men and women and a WC cutoff near 75–80 cm for women and 80–85 cm for men [15-17,30-33]. 

We found that though the Uighur population belonged to Asian population, the appropriate BMI cutoffs were slightly higher with the WHO definition which was based on the western population. Beside the ethnics differences, the main reason may be that the WHO definition was published nearly a decade ago, the prevalence among adults had increased by nearly 50% during the 1980s and 1990s [3], nearly 3 times of adults are classified as overweight or obese now compared with those 40 years ago [3-5]. Thus, as the growing prevalence of obesity worldwide [34,35], the BMI cutoffs may have some minor changes when compared with the definition 15 years ago.

We also found that the WC cutoffs in Uighur women were the same as the Uighur men. According to the WHO criteria, the WC cutoff point for men is 10 cm greater than that for women. It was reported that a cutoff value for men was 5 cm greater than for women among Korean adults [21]. One study in Chinese adults aged 35–74 years suggested the same WC value, 80 cm, for both men and women as the cutoff point for central obesity in identifying cardiovascular risk factors [20]. Our study was consistent with the results based on the Chinese adults. One Japanese study reported the WC cutoff point for women was consistent with our results [36]. They reported that the WC values of >85 cm for men and >90 cm for women were associated with a remarkable increase in the risk for cardiovascular disease. The reason why the WC cutoffs in Uighur women were at high levels was still unclear. Since most Uighur women become fat after giving birth to their children. The postnatal hormonal changes may provide beneficial effects to such subjects. 

The present study has several strengths. This is the first representative sample of the general adult Uighur population. Thus, these results can be generalized to the full adult population of Uighurs aged above 35 years. Additionally, we provided information for a wide range of BMI and waist circumference values, stratified by sex. The limitation of this study is its cross-sectional sample population. Future studies can use the BMI and waist circumference cutoffs suggested here to study the associated risk factors and intervention of overweight and obesity in a representative sample of the adult Uighur population [37,38]. 

In conclusion, this study showed a BMI value of 26 and a waist circumference value of 90 cm in both men and women as appropriate cutoffs in the identification of high-risk Uighur patients. The continuous relation between cardiovascular disease risk factors and BMI and waist circumference were documented here. This study enriched the limited data on the appropriate definition of overweight for immediate use in screening among Uighur population. 
